# Zinc Oxide Nanoparticles Biosynthesized by *Eriobotrya japonica* Leaf Extract: Characterization, Insecticidal and Antibacterial Properties

**DOI:** 10.3390/plants12152826

**Published:** 2023-07-31

**Authors:** Esraa Hamdy, Abdulaziz A. Al-Askar, Hamada El-Gendi, Wael M. Khamis, Said I. Behiry, Franco Valentini, Kamel A. Abd-Elsalam, Ahmed Abdelkhalek

**Affiliations:** 1 Plant Protection and Biomolecular Diagnosis Department, Arid Lands Cultivation Research Institute, City of Scientific Research and Technological Applications, Alexandria 21934, Egypt; esraah752@gmail.com; 2 Department of Botany and Microbiology, College of Science, King Saud University, P.O. Box 2455, Riyadh 11451, Saudi Arabia; aalaskara@ksu.edu.sa; 3 Bioprocess Development Department, Genetic Engineering and Biotechnology Research Institute, City of Scientific Research and Technological Applications, New Borg El-Arab City 21934, Egypt; elgendi1981@gmail.com; 4 Plant Protection Research Institute, Agriculture Research Center, Al-Sabhia, Alexandria 21616, Egypt; waelmkhamis2019@yahoo.com; 5 Agricultural Botany Department, Faculty of Agriculture (Saba Basha), Alexandria University, Alexandria 21531, Egypt; said.behiry@alexu.edu.eg; 6 Istituto Agronomico Mediterraneo di Bari, Via Ceglie 9, Valenzano, 70010 Bari, Italy; valentini@iamb.it; 7 Plant Pathology Research Institute, Agricultural Research Centre, Giza 12619, Egypt

**Keywords:** ZnO-NPs, *Eriobotrya*, FTIR, insecticidal, rice weevil, red flour beetle, stored products, bactericidal activity

## Abstract

Zinc oxide nanoparticles (ZnO-NPs) have gained significant attention in nanotechnology due to their unique properties and potential applications in various fields, including insecticidal and antibacterial activities. The ZnO-NPs were biosynthesized by *Eriobotrya japonica* leaf extract and characterized by various techniques such as UV–visible (UV–vis) spectrophotometer, X-ray diffraction (XRD), scanning electron microscopy (SEM), Fourier transform infrared spectroscopy (FTIR), dynamic light scattering (DLS), and zeta potential analysis. The results of SEM revealed that NPs were irregular and spherical-shaped, with a diameter between 5 and 27 nm. Meanwhile, DLS supported that the measured size distributions were 202.8 and 94.7 nm at 11.1° and 90.0°, respectively, which supported the polydisperse nature of NPs, and the corresponding zeta potential was −20.4 mV. The insecticidal activity of the produced ZnO-NPs was determined against the adult stage of coleopteran pests, *Sitophilus oryzae* (Linnaeus) (Curculionidae) and *Tribolium castaneum* (Herbst) (Tenebrionidae). The LC_50_ values of ZnO-NPs against adults of *S. oryzae* and *T. castaneum* at 24 h of exposure were 7125.35 and 5642.65 μg/mL, respectively, whereas the LC_90_ values were 121,824.56 and 66,825.76 μg/mL, respectively. Moreover, the biosynthesized nanoparticles exhibited antibacterial activity against three potato bacterial pathogens, and the size of the inhibition zone was concentration-dependent. The data showed that the inhibition zone size increased with an increase in the concentration of nanoparticles for all bacterial isolates tested. The highest inhibition zone was observed for *Ralstonia solanacearum* at a concentration of 5 µg/mL, followed by *Pectobacterium atrosepticum* and *P. carotovorum*. Eventually, ZnO-NPs could be successfully used as an influential agent in pest management programs against stored-product pests and potato bacterial diseases.

## 1. Introduction

The growing global population, degradation of agricultural lands by pollution with recalcitrant chemicals, and challenges brought about by climate change drive the need for crop productivity and improved food quality [1,2]. Over the past few decades, there has been an increase in the pace and enhancement of agricultural production through various means [3]. Plant diseases result in substantial agricultural losses and hinder progress. Among others, insect and bacterial plant infections pose a significant threat to plant biosecurity, resulting in substantial agricultural losses on a global scale.

The rice weevil, *Sitophilus oryzae* (Linnaeus) (Coleoptera: Curculionidae), and the red flour beetle, *Tribolium castaneum* (Herbst) (Coleoptera: Tenebrionidae), are dangerous pests of cereal grains and their by-products [4,5]. Rice weevil, *Sitophilus oryzae* (Linnaeus), is ecumenical and has aggravating damages with almost 65% loss in wheat seeds under medium-term storage conditions, or even 80% under long-term storage conditions [6,7]. Furthermore, adults and larvae of the red flour beetles have active feeding on stored foodstuffs, for instance, dry fruits, pulses, bran, coat, germ, grain dust, and prepared cereal foods [8]. The development of *T. castaneum* (Herbst) progeny has a preference for the genotypes of wheat more than rice and maize. The genotypes of these stored products could not reveal a complete resistance to *T. castaneum* (Herbst) infestation. Hence, the highest weight losses occur along the 90th days of exposure in all the genotype products [9,10,11]. Even though several conventional insecticides from different chemical groups are commonly used to prevent the loss of weight of stored grains and maintain their quality, the residual effect of conventional insecticides may cause food contamination. Numerous studies have found that resistant strains of insects have developed due to the overuse of conventional insecticides [12]. Additionally, plant pathogenic organisms are a group of microbial pathogens that hold immense significance and have a global distribution [13].

According to estimates, approximately 150 species out of the 7100 recognized bacteria have been identified as causative agents of diverse plant diseases [14]. *Pectobacterium carotovorum* and *P. atrosepticum* are Gram-negative, rod-shaped bacteria that belong to the family Enterobacteriaceae. They are plant pathogens known for causing soft rot or black-leg diseases which are characterized by the decomposition of plant tissues in a wide range of crops, including potatoes, carrots, onions, and other vegetables. The affected tissues have an unpleasant odor due to the production of volatile compounds by the bacteria. They produce several enzymes that contribute to their pathogenicity [13]. The pectin-rich middle lamella, which is responsible for maintaining the structural integrity of plant cells, is degraded by bacterial pectolytic enzymes. This phenomenon results in the separation of cells and the infliction of harm to the plant. The transmission of the ailment can occur through various means, including water, insects, or implements such as sickles [13,15,16,17]. *Ralstonia solanacearum* is a bacterial pathogen that causes a devastating disease known as bacterial wilt in many plants, including potatoes. The disease caused by *R. solanacearum* is commonly referred to as “brown rot” or “bacterial wilt.” When *R. solanacearum* infects potatoes, it can cause significant damage to the plants and lead to yield losses. The bacterium colonizes the xylem vessels, which are responsible for transporting water and nutrients throughout the plant. As a result, the bacteria block the vessels, impeding the flow of water and causing wilting and eventual death of the potato plants. Symptoms of *R. solanacearum* infection in potato plants include wilting and yellowing of the leaves, which typically starts with a single branch or stem and progresses to the entire plant. The bacteria can also cause vascular discoloration in the stem and tubers, leading to brown rot and decay [14,18]. At present, the main method commonly utilized for disease management is to apply extensive pesticides [19].

The utilization of dangerous agrochemicals to manage pathogens is often associated with a reduction in crop quality, significant health concerns for crop consumers, and ultimately, the accumulation of these chemicals in water sources, which exacerbates environmental challenges [20,21,22]. Therefore, contemporary techniques such as nanoparticles are imperatively required to supersede conventional chemical insecticides [23,24,25,26,27]. Nanomaterial applications were introduced as one of the great promises of technology in the field of plant protection [28]. Moreover, many attempts have been carried out to implement more NPs in biological applications, for instance, zinc oxide nanoparticles (ZnO-NPs). Zinc oxide nanoparticles have an outstanding role in enhancing agrochemical industries, besides their insecticidal and antimicrobial activities. Nowadays, green biogenic synthesis of ZnO-NPs are easily applied via plant extracts as a quite safe and ecofriendly method that can alter the chemical synthesis [27,29].

Generally, metal oxide nanoparticles (NPs) are noteworthy in the realm of antibacterial agents due to their catalytic inhibitory activity. The antimicrobial mechanisms of these agents are contingent upon various parameters, including their appearance, structure, and amount [30,31]. Moreover, Zanet et al. [32] conducted trials utilizing zinc oxide nanoparticles (NPs) on the reference cell *Saccharomyces cerevisiae* to determine the primary mechanism of action. Their findings suggest that the impact of ZnO-NPs is contingent upon their composition and dosage. Zinc oxide nanoparticles can be synthesized through various methods, including chemical precipitation, salt reduction, the sol–gel technique utilizing an acetate precursor, and sonochemical synthesis. Nevertheless, diverse synthetic routes produce zinc oxide particles exhibiting distinct morphologies and sizes [33,34,35]. Hence, the mechanism of action of these entities, along with their interplay with various cellular structures, may exhibit a considerable variability. Zinc oxide (ZnO) is classified as a transition metal oxide and a semiconductor. It possesses significant binding energy, which contributes to its highly oxidative nature. This property has been reported in the literature [36]. The process results in the generation of reactive oxygen species, which serves as the mechanism of bactericidal activity. Furthermore, an additional mechanism of action that exhibits bactericidal properties involves the liberation of zinc ions (Zn^2+^), which inflict harm upon the cellular membrane and potentially impede certain metabolic pathways [37]. Further investigations on the antibacterial mode of action of ZnO-NPs can significantly enhance our understanding of potential bacterial resistance mechanisms and aid in optimizing contact duration and effective inhibition measures.

Therefore, the present investigation aimed to employ an aqueous extract derived from *E. japonica* leaves as a stabilizing agent to accomplish the ecofriendly biosynthesis of ZnO-NPs, which were subjected to various analytical techniques and evaluated for their insecticidal and bactericidal activities against several plant pathogens. 

## 2. Results and Discussion

### 2.1. UV–vis and X-ray Diffraction (XRD) Analysis

**Figure 1:**
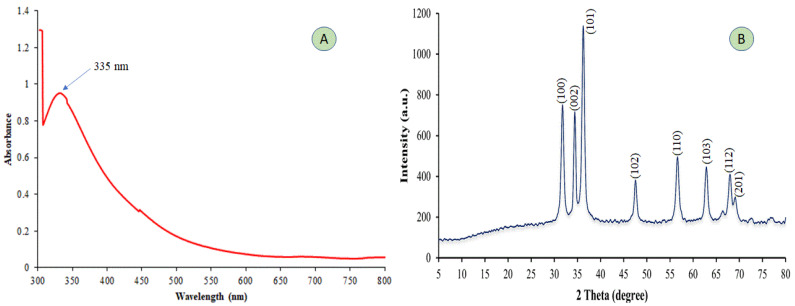
UV–vis spectral analysis (**A**) and X-ray diffraction (XRD) spectrum of synthesized ZnO-NPs (**B**).

The UV–visible absorption spectra of the synthesized zinc oxide nanoparticles were analyzed within the wavelength range of 300–800 nm, as depicted in Figure 1A. The majority of ZnO-NPs exhibit a surface plasmon resonance (SPR) band within the range of 220–380 [38,39,40]. This phenomenon is attributed to the stimulation of unbound electrons. As depicted in Figure 1A, the surface plasmon resonance (SPR) value of ZnO-NPs was determined to be 335 nm, consistent with previous research findings [41,42]. In another study conducted by Nazir et al. [43], the peak of green-synthesized ZnO-NPs using *E. japonica* leaf extract was detected at 375 nm. Figure 1B displays the XRD pattern of ZnO-NPs that were synthesized through the use of *E. japonica* leaf extract. The XRD data were analyzed, and the values obtained at 31.83, 34.32, 36.35, 47.34, 55.99, 62.83, 67.91, and 69.18 were assigned to the (100), (002), (101), (102), (110), (103), (112), and (201) crystallographic planes, respectively. The ZnO wurtzite structure (JCPDS card number 01-089-1397) was found to be consistent with the XRD peaks of biosynthesized ZnO-NPs by *E. japonica* plant extract [43,44,45]. In the same line, all the diffraction peaks were successfully indexed with the standard (JCPDS # 36–1451) [46]. The presence of sharp and narrow peaks suggests that the ZnO nanoparticles synthesized through biosynthesis exhibited a high degree of crystallization. The XRD findings in this study correspond with those reported by Shabaani et al. [47], Ali et al. [48], and Stan et al. [49]. Shabaani et al. [47] synthesized ZnO-NPs with *Eriobotrya japonica* seed extract; Ali et al. [48], synthesized ZnO nanoparticles using *Aloe vera* leaf extract; and Stan et al. [49] employed *Allium cepa* plant extract for the biosynthesis of nanoparticles. All studies observed crystalline particle sizes. Furthermore, it was observed that the ZnO nanoparticles were devoid of any impurities, as evidenced by the absence of X-ray diffraction peaks characteristic of substances other than zinc oxide [50,51].

### 2.2. Scanning Electron Microscopy Analysis

**Figure 2:**
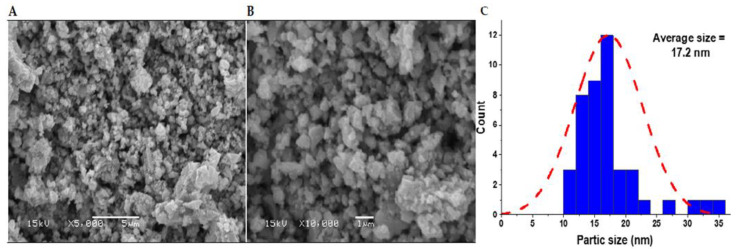
The scanning electron microscopy (SEM) of the prepared ZnO-NPs with corresponding particle size was retrieved through ImageJ software (**A**–**C**).

The surface morphology of the green-prepared ZnO-NPs was elucidated through SEM analysis. The results (Figure 2A,B) revealed irregular spherical-shaped NPs most likely to be hexagonal shapes that agglomerated into large network structures. The results follow Abdelmigid et al. who reported spherical and hexagonal ZnO-NPs prepared from *Punica granatum* peel and coffee ground extracts [52]. The particle size was evaluated through ImageJ software and revealed different-sized NPs from 10 to 35 nm with a 17.2 nm average size for the prepared ZnO-NPs (Figure 2C), which followed other green-synthesized ZnO-NPs from *Sambucus ebulus* leaf extract [53] and onion extract [54]. The findings of this study align with earlier research that employed ecofriendly methods (involving plant extracts) to produce variously shaped ZnO nanoparticles at the nanoscale. For instance, one such study used *Ziziphus jujuba* fruit extract as a synthesis medium for ZnO nanoparticles, resulting in spherical particles ranging between 21 and 37 nm in size [55]. Likewise, another investigation employed *Ziziphus nummularia* leaf extract to generate ZnO nanoparticles with sizes varying from 12.47 to 26.97 nm, which also exhibited a spherical morphology [56]. In the same line, particle sizes ranging from 15 to 18 nm for the resulting ZnO nanoparticles were reported in previous studies [47,48,49].

### 2.3. FTIR Analysis

**Figure 3:**
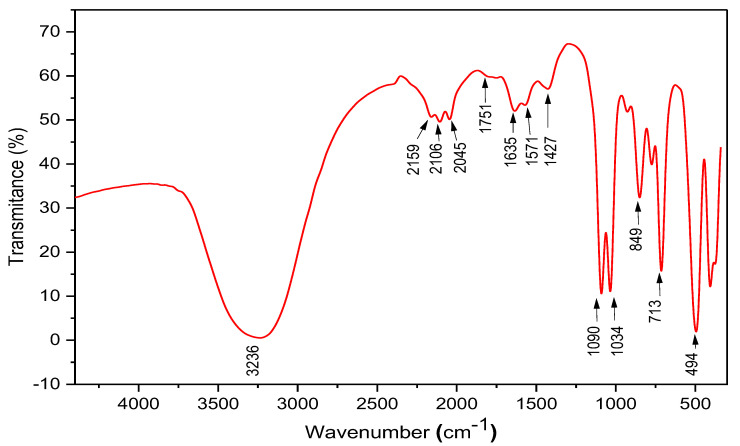
The FTIR analysis for evaluating functional groups in the green-prepared ZnO-NPs.

The functional groups in the prepared ZnO-NPs were analyzed through FTIR as indicated in Figure 3. The strong broad vibration at 3236 cm^−1^ indicated the presence of –OH groups [57]. Additionally, the small bands detected from 2159 to 2045 cm^−1^ indicated the stretching vibration of alkyne (–C≡C–) and nitrile (–C≡N) groups [58]. The small band detected at 1751 cm^−1^ indicated the stretching vibration of carbonyl groups (C=O) of ketones, aldehydes, and unsaturated esters [58]. Furthermore, the stretching vibration of carbonyl groups (C=O) was indicated by a vibration band at 1635 cm^−1^ [59,60], whereas the bands at 1571 and 1460 cm^−1^ indicated the stretching vibration of CH2 (methylene) from protein and C–C from aromatic groups, respectively [52]. Additionally, the strong bands detected at 1090–1034 cm^−1^ confirmed the stretching vibration of C–O groups related to alcohols and esters or the stretching vibration of C-N groups from carboxylic acids or aliphatic amines [61]. Other bands were detected through the FTIR analysis, as indicated by bands at 849, 713, and 494 cm^−1^. The band around 900–400 cm^−1^ is usually attributed to the Zn–O vibration, which confirmed the good crystallinity of the prepared NPs [52,62,63]. The various functional groups detected through the FTIR analysis in the green-synthesized ZnO-NPs could be attributed to an organic layer surrounding the NPs, resulting from prepared EJE [43,52]. As per the literature, the green-synthesized NPs are usually coated with an organic layer retrieved from the reducing solution related to flavonoids and phenolics molecules and claimed to enhance the ZnO-NPs or other NPs’ stability and bioactivity [47,52,53,64].

### 2.4. Particle Size Distribution and Zeta Potential Evaluation

**Figure 4:**
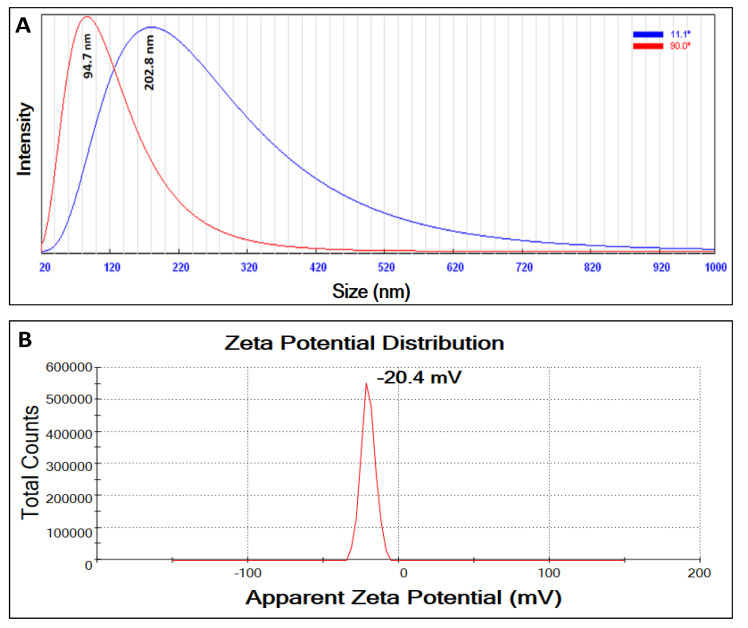
The particle size distribution of the prepared ZnO-NPs at two different angles 11.1° and 90° (**A**) and the corresponding zeta potential (**B**).

Dynamic light scattering (DLS) is widely accepted for determining nanoparticle size distribution [65]. In the current analysis, the particle size distribution was evaluated at two light scattering angles, 11.1° and 90.0°. The measured size distributions were 202.8 and 94.7 nm at 11.1° and 90.0°, respectively (Figure 4A). The indicated sizes were slightly higher than the SEM analysis results, which could be attributed to the polydisperse nature of the prepared ZnO-NPs. It is claimed that polydisperse NPs have a non-homogenous distribution that is usually indicated by higher particle sizes in light-scattering approaches [66]. Additionally, the coated organic layer on the NPs’ surface could also interfere with the particle size results, as reported by [52,67]. Herein, the measured polydispersity index of the green-synthesized ZnO-NP was 0.364, which is much lower than 0.7. Generally, samples with a polydispersity index < 0.7 are monodisperse, which indicates limited variation in their particle sizes [68]. As a result, the second reason for the organic coating is most likely to explain why the size increased in the DSL results when compared to the SEM results.

Furthermore, the surface charge of the prepared ZnO-NPs was evaluated through a Zetasizer. The results (Figure 4B) indicated negatively charged ZnO-NPs with a zeta potential of about −20.4 mV. The stability and toxicity of NPs are directly influenced by the final surface charge [69]. The significant stability of negatively charged NPs in the liquid preparations is attributed to strong electrostatic repulsion among particles [70]. Additionally, negatively charged NPs are safer for biological systems compared to positively charged ones that tend to attach to negatively charged cell envelopes (cell walls and cell membranes) and hence increase their toxicity [69,71]. As per the literature, NPs with lower zeta potentials ≥−30 mV are more stable and less toxic, which confirmed the stability and safety of the prepared ZnO-NPs in the current study [72]. The net negative charge in the prepared ZnO-NPs could be attributed to negatively functional groups such as hydroxyl and carboxylic groups coating the particles’ surfaces, as indicated in the FTIR results [64].

### 2.5. Toxicity of Zinc Oxide Nanoparticles

The entomo-toxicity of ZnO-NPs at LC_50_ and LC_90_ by fumigant application against the adult stages of *S. oryzae* (Linnaeus) and *T. castaneum* (Herbst) after 24 h of exposure were examined in the range of the foregoing concentrations (Table 1). Data obtained showed that the LC_50_ values of ZnO-NPs against adults of *S. oryzae and T. castaneum* were 7125.35 and 5642.65 μg/mL, respectively, whereas the LC_90_ values were 121,824.56 and 66,825.76 μg/mL, respectively. Both tested insects showed a gradual increase in their mortality response consonant with the increase in ZnO-NPs’ concentrations. According to these values, adults of both insects were susceptible to ZnO-NPs with no significant differences between their LC_50_ and LC_90_ values.

Our obtained data of the LC_50_ and LC_90_ values throughout the fumigant toxicity test came following the previous investigation conducted by Haroun et al. [73], who found that ZnO-NPs exhibited a significant toxic effect against *S. oryzae* (Linnaeus) at the highest concentration, but on the contrary, *T. castaneum* (Herbst) showed a high resistance. Additionally, Ibrahim et al. [74] demonstrated that the increases in the mortality of the adult stage of *S. oryzae* were realized on treated wheat grains by green-synthesized ZnO-NPs from pomegranate peel extract along the exposure intervals and at gradual concentrations. Further investigations on ZnO-NPs showed an observed effectiveness in controlling *T. castaneum* (Herbst), which may be qualified to be introduced in the future in integrated pest management [75].

### 2.6. ZnO-NPs Effect on Bacterial Strains

**Figure 5:**
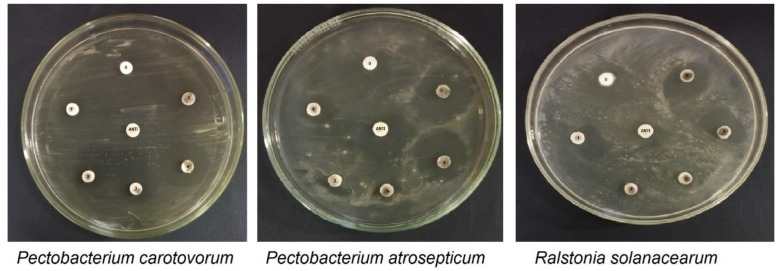
Shows the effect of *Eriobotrya japonica*-derived zinc oxide nanoparticles (ZnO-NPs) on various bacterial isolates.

The data presented in Table 2 show the response of different bacterial isolates to synthesized ZnO-NPs using the disc diffusion method. The inhibition zones (mm) were recorded at different concentrations (µg/mL) of exposure of NPs (Figure 5). The results demonstrate that the synthesized ZnO-NPs have an inhibitory effect on the growth of all tested bacterial isolates.

At a concentration of 1 µg/mL, *P. carotovorum* showed the lowest inhibition zone (8.00 mm) while *R. solanacearum* showed the highest inhibition zone (13.67 mm). As the concentration of ZnO-NPs increased, the inhibition zones also increased for all three bacterial isolates. At the highest concentration tested (5 µg/mL), all three bacterial isolates showed the highest inhibition zone, with *R. solanacearum* exhibiting the highest inhibition zone (23.00 mm), followed by *P. atrosepticum* (21.67 mm), and *P. carotovorum* (17.00 mm). The negative control (no ZnO-NPs) showed no inhibition zones for any of the bacterial isolates. The positive control, Augmentin (10 µg/disc), exhibited the highest inhibition zone for *P. atrosepticum* (25.00 mm), followed by *R. solanacearum* (19.00 mm) and *P. carotovorum* (13.33 mm). Our resulting data suggest that synthesized ZnO-NPs have an inhibitory effect on the growth of tested bacterial isolates and that this effect is concentration-dependent. The results also indicate that the ZnO-NPs have a greater inhibitory effect on *R. solanacearum* compared to *P. carotovorum* and *P. atrosepticum*, which support the strain-dependent activity of the prepared ZnO-NPs.

*Eriobotrya japonica*-leaf-extract-mediated zinc oxide nanoparticles show more significance against plant bacterial pathogens such as *P. carotovorum*, *P. atrosepticum*, and *R. solanacearum* than the reported values of Ag-NPs mediated by *Ficus sycomorus* leaf extract, as measured by inhibitory zone values [76]. Earlier generated ions [77,78,79] have also demonstrated a comparable suppression of ZnO-NPs generated by different methods against Gram-negative bacteria. Nevertheless, the IC_100_ values may vary slightly due to variations in nanoparticle fabrication, resulting in distinct features for each. In general, the outcomes exhibited a narrow margin of difference. The ZnO-NPs were successfully synthesized in our investigation, and their reduced size (17.2 nm) improved their sensitivity to act as antimicrobial agents, which aligns with findings from prior studies showing that smaller particle sizes of Ag-NPs had greater surface areas and an increased sensitivity to antimicrobial agents [80]. The synthesis of ZnO-NPs relies not only on their size but also on other parameters, such as interaction and stability with biological molecules. Due to their distinct chemical and physical features, nanoparticles of zinc oxide, generally ranging in size from 1 to 100 nm, are known for their efficacy against drug-resistant bacteria [81]. Nanomaterials with a higher surface area relative to volume ratio have been shown through investigations [81] to be more effective against bacteria due to their capacity to bind to and enter bacterial cells. Scientists utilize scanning electron microscopy or field emission scanning electron microscopy to examine the morphological alterations in bacteria caused by ZnO and measure the various mechanisms implicated. Notwithstanding the widespread investigation of the antibacterial properties of ZnO-NPs, the precise mechanism of their toxicity remains inadequately comprehended and a topic of debate. Additional elaboration is necessary to address various inquiries related to the range of antibacterial efficacy. Multiple modes of action, including the effects on the bacterial cell wall and membrane, contribute to the antibacterial activities of ZnO-NPs against *Escherichia coli*, *Staphylococcus aureus*, and *Pseudomonas aeruginosa* [81]. Protein and DNA functions, which are essential for many physiological activities including electron transport, protein synthesis, cell permeability, and DNA replication, the discharge of antimicrobial ions, mainly in the form of Zn^2+^ ions [82,83], and the production of reactive oxygen species (ROS) [84,85], has also been shown to be influenced by ZnO-NPs [86,87,88]. The mechanism of toxicity may exhibit variability across different media owing to the presence of diverse components and the physicochemical attributes of ZnO-NPs, which can potentially impact the dissolved zinc species [83].

These results set a precedent for the widespread use of ZnO-NPs as an efficient control agent for soft rot disease management. ZnO-NPs may be synthesized at low cost and investigated as a potential new antibacterial agent with the advent of nontoxic production technologies. ZnO-NPs were tested for their antibacterial properties against a variety of Gram-negative bacteria on agar plates, with positive results showing total bacterial growth suppression. For each bacterial strain and NP concentration, the level of inhibition varied [89]. ZnO-NPs have several benefits over traditional chemical antibacterial agents, particularly in agriculture, where antibiotic resistance is a major problem. The emergence of diverse resistance characteristics in different organisms over time is a problem with chemical antimicrobial agents since they rely on a particular binding between bacteria and the antimicrobial agent’s surface and metabolites. A possible alternative to traditional antibiotics for dealing with antibiotic-resistant microbes is metal nanoparticles such as ZnO-NPs, which are less likely to generate resistance in bacteria.

## 3. Materials and Methods

### 3.1. Preparation of ZnO-NPs through Eriobotrya japonica Leaf Extract (EJE)

The green-synthesized ZnO-NPs were prepared from *Eriobotrya japonica* leaf extract (EJE) using zinc acetate (Zn (CH_3_CO_2_)_2_, Sigma Aldrich, St. Louis, MO, USA) as a precursor. In brief, the plant leaves were collected from the Borg El-Arab City, Alexandria, Egypt, and morphologically identified as *Eriobotrya japonica* by scientists of the Plant Production Department, Faculty of Agriculture (Saba Basha), Alexandria University, Alexandria, Egypt. *Eriobotrya japonica* plant leaves were washed with dH_2_O several times and air-dried. Afterward, the dried *Eriobotrya japonica* leaves were ground to a fine powder and homogenized in a final ratio of 10 g to 100 mL of dH_2_O. The mixture was incubated at 50 °C for 2 h under shaking and then centrifuged at 5000 rpm for 10 min. About 10 mL of clear supernatant was added to 90 mL of 1M Zn (CH_3_CO_2_)_2_ solution. The generated ZnO-NPs (indicated by white precipitation) were separated by centrifugation, washed several times with dH_2_O, and dried at 50 °C (Figure S1).

### 3.2. Characterization of the Green Synthesized ZnO-NPs

The green-synthesized ZnO-NPs were characterized through different instrumental techniques. Scanning electron microscopy (SEM) was applied to elucidate the surface morphology and shape of the prepared ZnO-NPs using a JSM-6360 LA microscope (JEOL, Tokyo, Japan). An XRD-7000 (Shimadzu, Kyoto, Japan) diffractometer with a CuK radiation beam (λ = 0.154060 nm), 30 kV and 30 mA, and 10–80° in 2θ was used to generate X-ray diffraction (XRD) patterns. A UV–visible spectrophotometer (Shimadzu, Tokyo, Japan) was used to look for the ZnO-NPs. The reduction of Zn+ ions was confirmed by measuring at the UV-245 double beam (300–800 nm). The functional groups in the prepared ZnO-NPs were evaluated in the range of 400–4000 cm^−1^ through Fourier transform infrared spectroscopy (FTIR) using 8400 s Shimadzu FTIR (Japan) and the KBr-disc method. Furthermore, the Zetasizer ver. 6.2 (ZS, Malvern, Kassel, Germany) was applied to investigate the particle-size distribution, polydispersity index, and net surface charge (zeta-potential) of the prepared ZnO-NPs.

### 3.3. Insect Rearing Culture

#### 3.3.1. *Tribolium castaneum* (Herbst)

Two hundred adults of *T. castaneum* (Herbst) were introduced on untreated, sterilized wheat flour (500 g) in wide-mouthed glass jars (1 L). The neck of the jar was covered with a muslin cloth and fixed with a rubber band to prevent the insects from escaping. The rearing procedure was installed under laboratory conditions (30 ± 2 °C and R.H. 65–70%) in a Shel-lab incubator (model 15450, Sheldon Manufacturing, Inc., Cornelius, OR, USA) for 2 weeks to accomplish mating and oviposition activity. Then, the flour medium was separated by sieves from the infested flour, and adequate numbers of progeny adults were reused at the time of the toxicity experiment [90].

#### 3.3.2. *Sitophilus oryzae* (Linnaeus)

The same foregoing procedure of *T. castaneum* (Herbst) was followed in *S. oryzae* rearing except the used conditions and feeding medium were 28 ± 2 °C and R.H. 65 ± 5% and wheat grains (500 g), respectively [91].

### 3.4. Fumigant Toxicity Bioassay

The fumigant toxicity of ZnO-NPs on *S. oryzae* (Linnaeus) and *T. castaneum* (Herbst) adults in the presence of wheat (*Triticum aestivum* L.) grains were conducted with a slight modification from the method described by Germinara et al. [92]. A glass jar (0.5 L) was used as a set unit for the fumigation experiment. Wheat grains (50 g) were placed on the base of the jar, followed by 15 adults of one of the tested pests. The tested concentrations of ZnO-NPs ranged from 1000, 3000, 5000, 7000, and 10,000 µg/mL, with a zero concentration in the control treatment. Each concentration of ZnO-NPs was loaded in 100 μL portions onto a filter paper (Whatman No. 1, diameter 4.5 cm) with an Eppendorf pipette tip. The filter paper was hung in the center of the jar by one edge of a stainless-steel wire, while the other edge was attached to the undersurface of a screw cap. Then, the jar (fumigation unit) was tightly closed by the screw cap to allow the treated filter paper to release its ZnO-NPs internally for 24 h. The fumigation unit was replicated three times for each tested concentration of ZnO-NPs and the control as well. The toxicity test was carried out in the dark at 28 ± 2 °C and RH 60 ± 5% for 24 h of exposure. The treated insects in each tested concentration and the control were transferred to Petri dishes in fresh air for 12 h (sufficient to discover the alive individuals) before counting the dead individuals. Finally, the mortality percentages at 24 h of exposure were corrected by the formula of Abbott [93], and the lethal concentrations of LC_50_ and LC_90_ were calculated based on the probit analysis [94].

### 3.5. Bacterial Cultures and Antibacterial Study

The bacterial isolates of *Ralstonia solanacearum* (LN681200), *Pectobacterium atrosepticum* (MG706146), and *P. carotovorum* (MN598002) were used in this study. All the strains were cultured in Luria broth, which was purchased from Merck (Darmstadt, Germany), for a day, at a temperature of 30 °C, while being agitated at a rate of 200 revolutions per minute. The study employed the disc diffusion technique [95] to evaluate the antibacterial efficacy of the ZnO-NPs that were synthesized. The bacterial culture that was incubated overnight was standardized and subsequently inoculated onto agar plates to facilitate the growth of a homogenous microbial colony. Concentrations of 1, 2, 3, 4, and 5 µg/mL were utilized in the experimentation involving ZnO-NPs. Each clean disc was administered 20 µL of the aforementioned concentrations. Sterile, double-purified water and the antibiotic Augmentin (10 µg/disc) were utilized as the two types of controls to assess the antibacterial properties. Subsequently, the cultured plates were subjected to growth at a temperature of 30 °C for one day, following which the discs were mounted on the surface of the plates. The diameter of the zone of inhibition (mm) was assessed and contrasted with the control pairs. The trial was conducted three times.

### 3.6. Statistical Analysis

The biological experiments were conducted in triplicate, and the outcomes are presented as the mean value along with the corresponding standard error. An analysis of variance (ANOVA) was employed to assess whether there were any significant differences among the means of the data sets. Furthermore, chi square and Tukey post hoc tests were conducted to compare specific groups of interest, and statistical significance was defined as a *p*-value below 0.05.

## 4. Conclusions

The present investigation involved the synthesis of zinc oxide nanoparticles (ZnO-NPs) utilizing *Eriobotrya japonica* leaf extract and their efficacy evaluation against plant pathogens. The ZnO-NPs were characterized using various methods and exhibited a strong efficacy, resulting in notable decreases in the populations of *S. oryzae* (Linnaeus) and *T. castaneum* (Herbst) in stored grains. Moreover, the NPs were effective against the studied bacterial strains, *Ralstonia solanacearum*, *Pectobacterium atrosepticum*, and *P. carotovorum*. These findings suggest that synthesized NPs have the potential to serve as a protective agent for seeds, provided that appropriate safety precautions are taken during application.

## Figures and Tables

**Table 1:** Toxicity of the zinc oxide nanoparticles on adults of *Sitophilus oryzae* and *Tribolium castaneum* at 24 h of exposure under storage conditions.

Adult Insect Pest	Lethal Concentration (μg/mL)		95% Fiducial Limits (μg/mL)	Slope ± SE **	*χ^2^* ***	*df*	N ****
*Sitophilus oryzae*	LC_50_	7125.35	(4443.56–11,425.67)	2.23 ± 0.03	34.26	3	225
	LC_90_	121,824.56	(9303.46–1,595,237.78)				
*Tribolium castaneum*	LC_50_	5642.65	(3867.93–8231.64)	2.23 ± 0.03	48.44	3	255
	LC_90_	66,825.76	(10,529.88–424,096.07)				

** Standard error. *** Chi square. **** Total numbers of insect individuals submitted to the toxicity test.

**Table 2:** The recorded inhibition zone (mm) signifies the varying responses of different bacterial isolates to the synthesized zinc nanoparticles (ZnO-NPs) derived from *Eriobotrya japonica* leaf extract (EJE).

ZnO-NPs Concentration (µg/mL)	Bacteria Inhibition Zone (mm)		
	*Pectobacterium carotovorum*	*P. atrosepticum*	*Ralstonia solanacearum*
1	8.00 e	9.33 d	13.67 c
2	10.33 de	9.67 d	11.33 c
3	13.67 bc	14.33 c	18.00 b
4	16.67 ab	21.00 b	19.33 b
5	17.00 a	21.67 b	23.00 a
Negative control (sterile distilled water)	0.00 f	0.00 e	0.00 d
Augmentin 10 µg/disc	13.33 cd	25.00 a	19.00 b

If the articles adjacent to the information in every column differ, there is a 0.01 probability of indicating a substantial dissimilarity in the data.

## Data Availability

To access the experimental data that support the findings of this study, interested individuals can contact the corresponding authors (A.A. and S.B.) directly and request the data.

## Supplementary Materials

The following supporting information can be downloaded at: https://www.mdpi.com/article/10.3390/plants12152826/s1, Figure S1: Synthesis of ZnO nanoparticles by *Eriobotrya japonica* leaf extract.
