# Supplementary figures and images for: Zinc Oxide Nanoparticles Biosynthesized by Eriobotrya japonica Leaf Extract: Characterization, Insecticidal and Antibacterial Properties
Source: Plants (Basel). 2023 Jul 31;12(15):2826. doi: 10.3390/plants12152826 (PMC10421472; doi:10.3390/plants12152826)

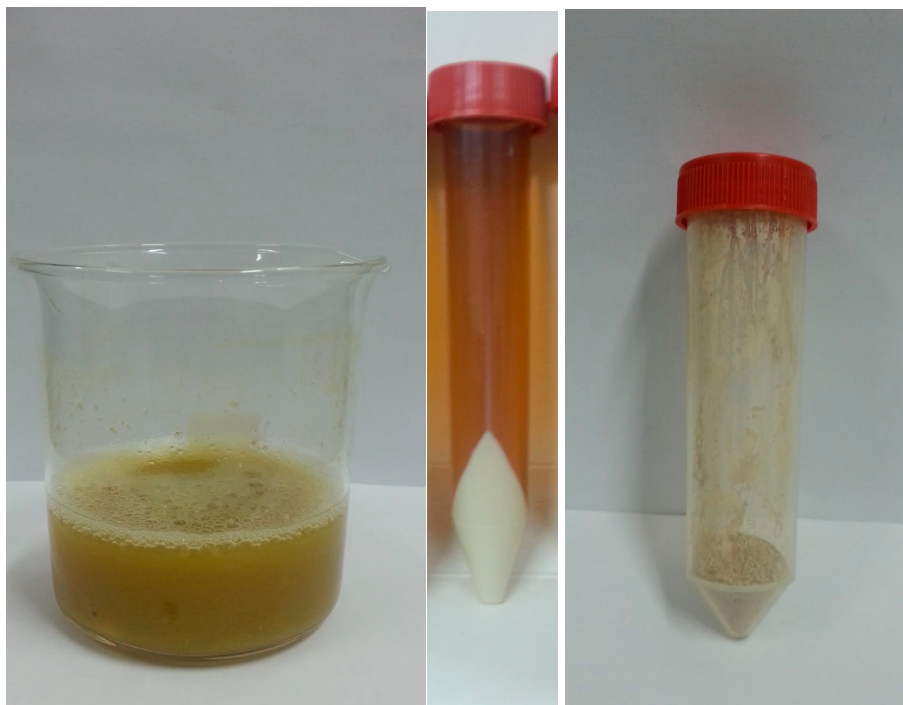

**Figure S1.** Synthesis of ZnO nanoparticles by *Eriobotrya japonica* leaves extract.

Supplement: Supplementary file 1 [file plants-12-02826-s001.zip › plants-2488831-supplementary.pdf]
